# Ultrasonic Assessment of Liver Fibrosis Using One-Dimensional Convolutional Neural Networks Based on Frequency Spectra of Radiofrequency Signals with Deep Learning Segmentation of Liver Regions in B-Mode Images: A Feasibility Study

**DOI:** 10.3390/s24175513

**Published:** 2024-08-26

**Authors:** Haiming Ai, Yong Huang, Dar-In Tai, Po-Hsiang Tsui, Zhuhuang Zhou

**Affiliations:** 1 Faculty of Science and Technology, Beijing Open University, Beijing 100081, China; aihm@bjou.edu.cn; 2 Department of Biomedical Engineering, College of Chemistry and Life Science, Beijing University of Technology, Beijing 100124, China; huangy@emails.bjut.edu.cn; 3 Department of Gastroenterology and Hepatology, Chang Gung Memorial Hospital at Linkou, Chang Gung University, Taoyuan 333423, Taiwan; tai48978@cgmh.org.tw; 4 Department of Medical Imaging and Radiological Sciences, College of Medicine, Chang Gung University, Taoyuan 333323, Taiwan; 5 Division of Pediatric Gastroenterology, Department of Pediatrics, Chang Gung Memorial Hospital at Linkou, Taoyuan 333423, Taiwan; 6 Liver Research Center, Chang Gung Memorial Hospital at Linkou, Taoyuan 333423, Taiwan; 7 Research Center for Radiation Medicine, Chang Gung University, Taoyuan 333323, Taiwan

**Keywords:** ultrasound radiofrequency signal, convolutional neural network, deep learning, liver fibrosis, liver region segmentation

## Abstract

The early detection of liver fibrosis is of significant importance. Deep learning analysis of ultrasound backscattered radiofrequency (RF) signals is emerging for tissue characterization as the RF signals carry abundant information related to tissue microstructures. However, the existing methods only used the time-domain information of the RF signals for liver fibrosis assessment, and the liver region of interest (ROI) is outlined manually. In this study, we proposed an approach for liver fibrosis assessment using deep learning models on ultrasound RF signals. The proposed method consisted of two-dimensional (2D) convolutional neural networks (CNNs) for automatic liver ROI segmentation from reconstructed B-mode ultrasound images and one-dimensional (1D) CNNs for liver fibrosis stage classification based on the frequency spectra (amplitude, phase, and power) of the segmented ROI signals. The Fourier transform was used to obtain the three kinds of frequency spectra. Two classical 2D CNNs were employed for liver ROI segmentation: U-Net and Attention U-Net. ROI spectrum signals were normalized and augmented using a sliding window technique. Ultrasound RF signals collected (with a 3-MHz transducer) from 613 participants (Group A) were included for liver ROI segmentation and those from 237 participants (Group B) for liver fibrosis stage classification, with a liver biopsy as the reference standard (Fibrosis stage: F0 = 27, F1 = 49, F2 = 51, F3 = 49, F4 = 61). In the test set of Group A, U-Net and Attention U-Net yielded Dice similarity coefficients of 95.05% and 94.68%, respectively. In the test set of Group B, the 1D CNN performed the best when using ROI phase spectrum signals to evaluate liver fibrosis stages ≥F1 (area under the receive operating characteristic curve, AUC: 0.957; accuracy: 89.19%; sensitivity: 85.17%; specificity: 93.75%), ≥F2 (AUC: 0.808; accuracy: 83.34%; sensitivity: 87.50%; specificity: 78.57%), and ≥F4 (AUC: 0.876; accuracy: 85.71%; sensitivity: 77.78%; specificity: 94.12%), and when using the power spectrum signals to evaluate ≥F3 (AUC: 0.729; accuracy: 77.14%; sensitivity: 77.27%; specificity: 76.92%). The experimental results demonstrated the feasibility of both the 2D and 1D CNNs in liver parenchyma detection and liver fibrosis characterization. The proposed methods have provided a new strategy for liver fibrosis assessment based on ultrasound RF signals, especially for early fibrosis detection. The findings of this study shed light on deep learning analysis of ultrasound RF signals in the frequency domain with automatic ROI segmentation.

## 1. Introduction

Early detection of liver fibrosis is very important as it can progress to liver cirrhosis and even hepatocellular carcinoma [1]. Currently, a liver biopsy [2] still serves as the golden standard for staging liver fibrosis. However, it is invasive and may cause complications and sampling errors [3]. Therefore, noninvasive imaging methods for liver fibrosis staging are of high interest. Among the different medical imaging modalities, ultrasound imaging is frequently used in clinical practice because it is real-time, low-cost, and widely available.

B-mode ultrasound imaging has been mostly used for liver fibrosis assessment. However, B-mode ultrasound is qualitative because it is constructed from the logarithmic compression of the envelopes of ultrasound-backscattered radiofrequency (RF) signals and can be affected by post-processing parameters such as the dynamic range. Moreover, the most significant source of variability in B-mode ultrasound is the insufficiently trained users. The original ultrasound-backscattered RF signals contain more abundant information than a B-mode ultrasound and can be used to extract different quantitative ultrasound parameters from the frequency, phase, and statistical information of RF signals, which have been widely used for biological tissue characterization [4]. Acoustically, biological tissue can be modeled as an ensemble of small particles that scatter sound waves, i.e., scatterers. The interaction of the incident ultrasound waves with the scatterers is carried in the backscattered RF signals [5,6]. Such correlations between ultrasound RF signals and tissue scatterers can be utilized to characterize microstructural alterations in the tissue that are not evident on conventional B-mode ultrasound images [5].

Recently, deep learning analysis of ultrasound-backscattered RF signals has emerged for biological tissue characterization [7,8,9,10,11,12,13]. The abundant information contained in the ultrasound RF signals can be automatically extracted by convolutional neural networks (CNNs) to yield a large number of feature parameters. By contrast, for the quantitative ultrasound techniques [4,5,6], a specific mathematical or physical model needs to be used to extract one feature parameter each time, usually under specific model assumptions. Previous studies have demonstrated the capability of one-dimensional (1D) CNNs based on time-domain RF signals in characterizing hepatic steatosis [7,8], osteoporosis [10], glioma [13], and liver fibrosis [10,12]. The frequency-domain information of RF signals has also been used to build CNN models to assess hepatic steatosis [11]. However, the feasibility of 1D CNNs applied to frequency spectra of RF signals in characterizing liver fibrosis has not been explored. Furthermore, when analyzing the liver RF signals, the liver region of interest (ROI) corresponding to the liver parenchyma generally needs to be identified first, but manual segmentation of liver ROIs from B-mode ultrasound images has been used in previous studies [8,10,11,12]. Hence, there is a need for automatic segmentation of liver ROIs.

In this paper, we proposed a two-step method for assessing liver fibrosis using deep learning models applied to ultrasound RF signals. First, two-dimensional (2D) CNNs were employed for automatic segmentation of liver ROIs from B-mode ultrasound images reconstructed from the RF signals. Second, 1D CNNs were used to classify liver fibrosis stages based on frequency spectra (amplitude, phase, and power) of the segmented ROI signals. Inspired by the work of Sanabria et al. [11], we hypothesized that the frequency-domain information, i.e., frequency spectra, of ultrasound RF signals may be utilized to build 1D CNN models for liver fibrosis characterization. We also hypothesized that 2D CNN models based on reconstructed B-mode ultrasound images may be used for automatic liver ROI segmentation. To test the two hypotheses, ultrasound RF signals collected from 613 participants were included for liver ROI segmentation and those from 237 participants for liver fibrosis stage classification. Experimental results showed that the proposed method is feasible for automatic liver ROI segmentation and liver fibrosis stage classification. The novelty of this work consists of using both 2D and 1D CNNs for liver parenchyma detection and liver fibrosis characterization.

This paper is organized as follows. Section 2 describes the ultrasound RF signal collection and the proposed method. Section 3 presents the results of liver ROI segmentation and liver fibrosis stage classification. Discussion and conclusions are given in Section 4 and Section 5, respectively.

## 2. Materials and Methods

**Figure 1:**
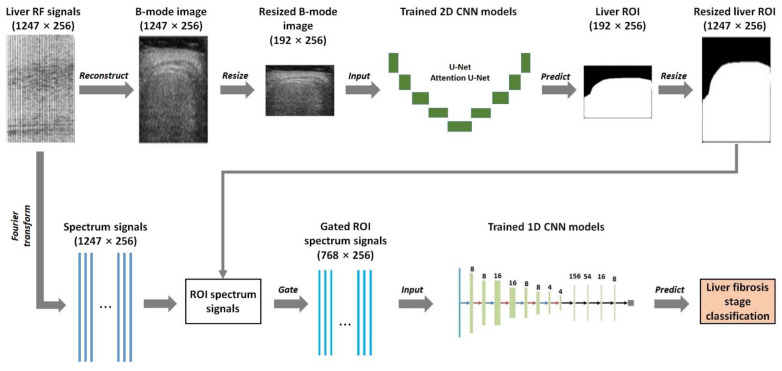
Flow chart of the proposed liver fibrosis stage classification method using deep learning models applied to ultrasound radiofrequency (RF) signals. First, two-dimensional (2D) convolutional neural networks (CNNs) were utilized for automatic liver region of interest (ROI) segmentation from B-mode ultrasound images reconstructed from RF signals. Second, one-dimensional (1D) CNNs were employed to classify liver fibrosis stages based on the frequency spectra (amplitude, phase, and power) of the segmented ROI signals. The white pixels in the liver ROI images represent the detected liver region whose values were 1, and the black pixels represent the detected non-liver region whose values were 0. The ROI spectrum signals were obtained by multiplying the spectrum signals by the resized liver ROI image. The size of the signals or the images is indicated as (*a* × *b*), where *a* is the sampling point number of a frame of signals or the height of an image, and *b* is the scan line number of the frame of signals or the width of the image.

Figure 1 shows the flow chart of the proposed liver fibrosis stage classification approach using deep learning models applied to ultrasound RF signals. The proposed approach was a two-step method. First, 2D CNNs were utilized for automatic liver ROI segmentation from B-mode ultrasound images reconstructed from the RF signals. In this study, two classical 2D CNNs, i.e., U-Net [14] and Attention U-Net [15], were used for liver ROI segmentation because U-Net and its extension networks, such as Attention U-Net, could yield fine segmentation for medical images, even with a small number of training samples. The B-mode images were obtained using the Hilbert transform and logarithmic compression and underwent data augmentation before training 2D CNN models. Binary images of liver ROIs were obtained using the trained 2D CNN models. The segmentation performance of U-Net and Attention U-Net models was compared, and the model with better segmentation performance was selected as the final 2D CNN model. Second, 1D CNNs were employed to classify liver fibrosis stages based on frequency spectra (amplitude, phase, and power) of the segmented ROI signals. The frequency spectra were obtained using the Fourier transform and underwent normalization and data augmentation before training 1D CNN models. Liver fibrosis stages were classified using the trained 1D CNN models.

This section is organized as follows. Section 2.1 describes the RF data collection. Section 2.2 describes the methodology of the 2D CNN-based liver ROI segmentation. Section 2.3 describes the methodology of the 1D CNN-based liver fibrosis stage classification. Section 2.4 describes the segmentation and classification performance evaluation metrics.

### 2.1. Clinical Data

The liver ultrasound RF signals included in this study were divided into two groups. Group A contained 613 cases of liver ultrasound RF signals, which were used for training and testing 2D CNN models for automatic liver ROI segmentation based on reconstructed B-mode images (Figure 1). Among them, 237 cases corresponded to adult liver fibrosis [16], 41 cases corresponded to pediatric liver fibrosis, 204 cases corresponded to adult hepatic steatosis, and 131 corresponded to pediatric hepatic steatosis. Group B contained 237 cases of ultrasound RF signals of adult liver fibrosis [16], which were used for training and testing 1D CNN models for liver fibrosis stage classification based on ROI spectrum signals (Figure 1).

The Institutional Review Board of Chang Gung Memorial Hospital at Linkou, Taiwan, approved the data collection. An informed consent form was signed by each participant or his/her guardian. The experiments were conducted following the approved guidelines. Ultrasound RF signals were collected using a clinical ultrasound scanner (Model 3000, Terason, Burlington, MA, USA) and a convex-array transducer with a 3 MHz central frequency and a 12 MHz sampling frequency. Each case of ultrasound RF signals was a frame of signals collected from one participant. Each frame of RF signals was composed of 256 scan lines, and each scan line had 1247 sampling points.

As the clinical reference standard for liver fibrosis staging, a liver biopsy and the METAVIR scoring system were used to semi-quantitatively classify liver fibrosis into 5 stages: F0–F4. F0 corresponded to no fibrosis, F1 corresponded to portal fibrosis with no septa, F2 corresponded to portal fibrosis with few septa, F3 corresponded to bridging fibrosis with many septa, and F4 corresponded to cirrhosis (nodular stage). In this study, the METAVIR scores were used as the reference standard for classifying liver fibrosis stages in Group B with the proposed method. The number of participants with different stages of liver fibrosis was scored as F0 = 27, F1 = 49, F2 = 51, F3 = 49, and F4 = 61.

### 2.2. Liver ROI Segmentation Using B-Mode Image-Based 2D CNNs

#### 2.2.1. B-Mode Image Reconstruction from Ultrasound RF Signals

For Group A, B-mode images were reconstructed from ultrasound RF signals. First, the Hilbert transform was used to detect the envelopes of a frame of RF signals. Second, logarithmic compression was conducted on the detected envelopes (dynamic range: 40 dB) to obtain a B-mode image. Note that no digital scan conversion was performed to reconstruct the B-mode images. Each B-mode image was sized 1247 pixels × 256 pixels (height × width). The reference standard of liver ROIs for the 2D CNN models was obtained by manual delineation of the reconstructed B-mode images by expert radiologists. Therefore, 613 B-mode images were obtained, corresponding to the 613 frames of RF signals in Group A. The B-mode image reconstruction was performed using MATLAB 2019 (MathWorks, Natick, MA, USA). Specifically, the MATLAB subroutine hilbert() was used for envelope detection.

#### 2.2.2. Data Augmentation for B-Mode Images

The 613 B-mode images were randomly divided on the participant level into a training set, a validation set, and a testing set in a ratio of 60%:20%:20%. Consequently, there were 367 B-mode images in the training set, 122 B-mode images in the validation set, and 124 B-mode images in the testing set. Data augmentation was performed to increase the number of samples in the training set and the validation set. The data augmentation included rotation and random cutting. The rotation angle was randomly set to 0°, 90°, 180°, or 270°. In addition, we adjusted the brightness, saturation, and contrast of the B-mode images to increase sample diversity. After data augmentation, the training set had 734 B-mode images, and the validation set had 244 B-mode images. Each B-mode image was resized to 192 pixels × 256 pixels (height × width) using bicubic interpolation to speed up 2D CNN training and to reduce the parameter size of the trained 2D CNN models.

#### 2.2.3. Network Structures of U-Net and Attention U-Net

**Figure 2:**
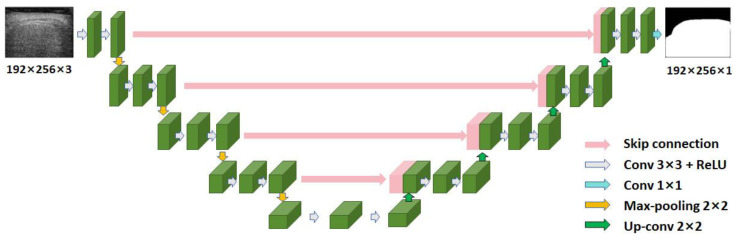
The network structure of U-Net. Conv: convolution; ReLU: rectified linear unit.

U-Net [14] is a deep-learning network specifically designed for medical image segmentation. Its structure was an improvement over the fully convolutional network [17]. Figure 2 shows the network structure of U-Net. U-Net had a topology structure of an encoder and a decoder, with skip connections between them. The input to U-Net was a 3-channel B-mode ultrasound image sized 192 pixels × 256 pixels (height × width). On the left side of the network was the encoder (Figure 2), which captured contextual information and extracted features. An encoder layer consisted of two 3 × 3 convolutional layers with a step of 1 and one 2 × 2 max-pooling layer with a step of 2. The output of the convolutional layer was batch-normalized and then activated using a rectified linear unit (ReLU) [18]. On the right side was the decoder (Figure 2) for precise positioning. A decoder layer consisted of two 3 × 3 convolutional layers with a step of 1. The up-sampling in the decoder was performed using transpose convolution. The decoder used the extracted features and the information provided by skip connections to restore the size of the image and to produce fine segmentation. The output of U-Net was a 1-channel liver ROI binary image sized 192 pixels × 256 pixels (height × width).

**Figure 3:**
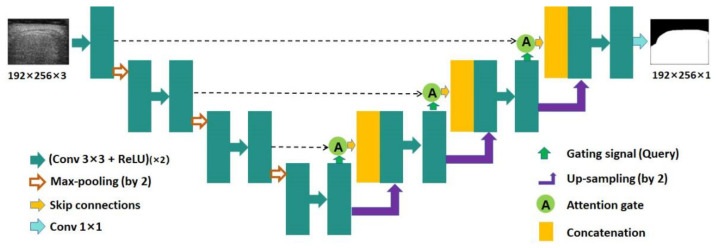
The network structure of Attention U-Net. Conv: convolution; ReLU: rectified linear unit.

Attention U-Net [15] was an extended network of U-Net [14], which incorporated the attention mechanism into U-Net. Figure 3 shows the network structure of Attention U-Net. The network structure of Attention U-Net was similar to that of U-Net. In Attention U-Net, the attention gate was integrated into the skip connection and up-sampling modules. Through the attention mechanism, the networks could suppress irrelevant information in the image and highlight important local features.

#### 2.2.4. 2D CNN Model Training and Testing

The 2D CNN (U-Net and Attention U-Net) models were trained and tested on a personal computer with an Intel(R) Xeon(R) W-2104 CPU @ 3.20 GHz, Nvidia Quadro P400 GPU, and 16 GB RAM. PyTorch (version 1.11.0) was used as the deep learning framework. The batch size and the number of epochs were set to 2 and 100, respectively. Adam [19] was used as the gradient optimizer, with an initial learning rate of 2 × 10^–4^ and betas of (0.9, 0.999). The loss function was the binary cross-entropy function [20]. After B-mode image segmentation, the detected liver ROI image was resized back to 1247 pixels × 256 pixels (height × width) using bicubic interpolation to match the size of the spectrum signals (Figure 1).

### 2.3. Liver Fibrosis Stage Classification Using ROI Spectrum Signal-Based 1D CNNs

#### 2.3.1. Frequency Spectrum Analysis of Ultrasound RF Signals

Quantitative ultrasound parameters could be obtained by extracting the spectral information of ultrasound-backscattered RF signals [21,22,23,24,25], which could be used to analyze tissue characteristics at different frequencies and provide a new perspective for tissue characterization. Therefore, we extracted three kinds of frequency spectrum information, i.e., the amplitude spectrum, phase spectrum, and power spectrum, from the ultrasound RF signals in Group B by performing the Fourier transform.

The amplitude spectrum of a signal is the amplitude distribution of the signal at different frequencies. Let **s** denote a scan line of ultrasound RF signals; the frequency spectrum of **s**, **S**_F_, was obtained as follows:

**S**_F_ = fft(**s**), (1)

where fft(.) denotes the fast Fourier transform. The amplitude spectrum, **S**_A_, was obtained as follows:

**S**_A_ = |**S**_F_|, (2)

The phase spectrum of a signal is the phase distribution of the signal at different frequencies. The phase spectrum, **S**_PH_, was obtained as follows:

**S**_PH_ = angle(**S**_F_), (3)

where angle(.) denotes the phase angle function.

The power spectrum of a signal is the energy distribution of the signal at different frequencies. The power spectrum, **S**_PW_, was obtained as follows:

**S**_PW_ = |**S**_F_|^2^/length(**S**_F_), (4)

where length(.) denotes the signal length function.

The MATLAB subroutine fft() was used for the fast Fourier transform. The MATLAB subroutine abs() was used to compute the amplitude spectrum. The MATLAB subroutine angle() was used to compute the phase angle.

#### 2.3.2. ROI Spectrum Signal Normalization

With the segmented liver ROI image by the 2D CNN models, the ROI spectrum signals were obtained by multiplying the spectrum signals sized 1247 points × 256 lines (axial × lateral) by the binary, resized liver ROI image sized 1247 pixels × 256 pixels (height × width) (Figure 1). Consequently, the pixel values in the non-liver region of the ROI spectrum signals were 0. A data normalization technique was introduced to normalize the ROI spectrum signals. Specifically, for a frame of ROI spectrum signals, **R**, the min-max normalization method was used:

**R**’ = (**R** − **R**_min_)/(**R**_max_ − **R**_min_) (5)

where **R**’ is the normalized ROI spectrum signals, and **R**_min_ and **R**_max_ are the minimum and maximum values of **R**, respectively. The MATLAB subroutine mapminmax()was used for spectrum signal normalization. In the following text, the normalized ROI spectrum signals were used where applicable.

#### 2.3.3. Data Augmentation for ROI Spectrum Signals

**Figure 4:**
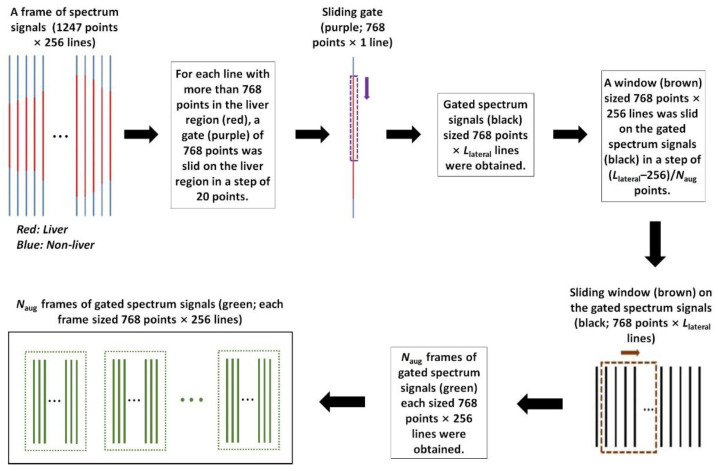
The flow chart of data augmentation for ROI spectrum signals. ROI: region of interest.

To address the unbalanced data distribution issue and avoid overfitting [26], a data augmentation method was employed for ROI spectrum signal augmentation before training the 1D CNN models. Take ≥F2 (i.e., binary classification between F0–F1 and F2–F4) for instance. The number of the ROI spectrum signals of F0–F1, *N*_01_, was less than that of F2–F4, *N*_24_. To avoid overfitting, the ROI spectrum signals of F0–F1 were augmented by a factor of *N*_aug_, *N*_aug_ = *N*_24_/*N*_01_. Figure 4 shows the flow chart of the data augmentation for ROI spectrum signals. The input was a frame of spectrum signals sized 1247 points × 256 lines (axial × lateral). For each line with more than 768 points in the liver region (indicated as the red solid lines in Figure 4), a gate of 768 points (indicated as the purple dashed line in Figure 4) was slid on the liver region in a step of 20 points. As a result, we obtained a frame of gated spectrum signals (indicated as the black solid lines in Figure 4) sized 768 points × *L*_lateral_ lines (axial × lateral). Next, a window (indicated as the brown dashed rectangle in Figure 4) sized 768 points × 256 lines (axial × lateral) was slid on the gated spectrum signals in a step of (*L*_lateral_ − 256)/*N*_aug_ to obtain *N*_aug_ frames of gated spectrum signals (indicated as the green solid lines in Figure 4); each frame sized 768 points × 256 lines (axial × lateral). Note that the sizes of the sliding gate (768 points × 1 line) and the sliding window (768 points × 256 lines) were experimentally set. After data augmentation, the spectrum signal samples were randomly divided on the participant level into a training set, a validation set, and a test set in accordance with a ratio of 80%:10%:10%.

#### 2.3.4. Network Structure of the 1D CNN

**Figure 5:**
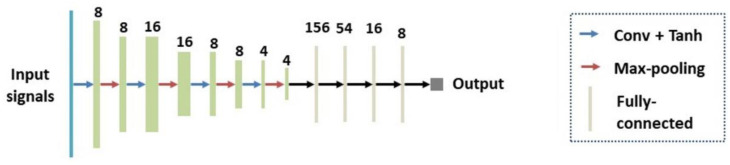
The network structure of the 1D CNN. Conv: Convolutional layers; 1D: one-dimensional; CNN: convolutional neural network.

Figure 5 shows the network structure of the 1D CNN for liver fibrosis stage classification. The 1D CNN had four 1D convolutional layers, four max-pooling layers, and four fully-connected layers. The features extracted by the convolutional and pooling layers were integrated into a single 1D feature vector. The 1D feature vector was taken as an input to the fully connected layer to output a prediction. Tanh(.) [27] was used as the activation function, which had a fast convergence speed and could effectively avoid oscillation of loss values.

#### 2.3.5. 1D CNN Model Training and Testing

In this study, four kinds of liver fibrosis stage classification experiments were conducted: (i) ≥F1, the binary classification between F0 and F1–F4; (ii) ≥F2, the binary classification between F0–F1 and F2–F4; (iii) ≥F3, the binary classification between F0–F2 and F3–F4; and (iv) ≥F4, the binary classification between F0–F3 and F4. The 1D CNN models were trained and tested for each of the three kinds of frequency spectra, i.e., amplitude, phase, and power, using the same computer and deep learning framework as the 2D CNN models. The batch size and the number of epochs were set to 256 and 100, respectively. Adam [19] was used as the gradient optimizer, with an initial learning rate of 2 × 10^–4^ and betas of (0.9, 0.999). The loss function was the cross-entropy function [20]. For a frame of ROI spectrum, signals sized 768 points × 256 lines in the test set, each line of ROI spectrum signals were input to the trained 1D CNN model, so 256 predictions were obtained. Let *n*_c_ and *n*_w_ denote the correct and wrong predictions, respectively. If the whole-frame prediction probability *p* = *n*_c_/(*n*_c_ + *n*_w_) was greater than 0.5, then the whole-frame prediction was determined as a correct classification.

### 2.4. Performance Evaluation Metrics

#### 2.4.1. Evaluation Metrics for Liver ROI Segmentation

To evaluate the performance of the 2D CNN models for liver ROI segmentation, six evaluation metrics were used: Jaccard similarity coefficient (JSC), Dice similarity coefficient (DSC), accuracy (ACC), sensitivity (SEN), precision (PRE), and specificity (SPE). Each metric had a value ranging from 0 to 1. A higher value corresponded to a better segmentation. The metrics were defined as follows:

(6) $$\mathrm{JSC}(A,B)=\frac{\left|A\cap B\right|}{\left|A\cup B\right|};$$

(7) $$\mathrm{DSC}(A,B)=\frac{2\left|A\cap B\right|}{\left|A\right|+\left|B\right|};$$

(8) $$\mathrm{ACC}=\frac{\left|TPP\right|+\left|TNP\right|}{\left|TPP\right|+\left|FPP\right|+\left|TNP\right|+\left|FNP\right|};$$

(9) $$\mathrm{SEN}=\frac{\left|TPP\right|}{\left|TPP\right|+\left|FNP\right|};$$

(10) $$\mathrm{PRE}=\frac{\left|TPP\right|}{\left|TPP\right|+\left|FPP\right|};$$

(11) $$\mathrm{SPE}=\frac{\left|TNP\right|}{\left|TNP\right|+\left|FPP\right|},$$

where *A* is the liver region predicted by the 2D CNN models, and *B* is the liver region manually delineated by human experts. *TPP* represents true positive pixels, *TNP* represents true negative pixels, *FPP* represents false positive pixels, and *FNP* represents false negative pixels.

#### 2.4.2. Evaluation Metrics for Liver Fibrosis Stage Classification

To evaluate the performance of the 1D CNN models for liver fibrosis stage classification, four metrics were used: ACC, SEN, SPE, and area under the receiver operating characteristic (ROC) curve (AUC) [28]. Each metric had a value ranging from 0 to 1. A higher value corresponded to a better classification. The metrics of ACC, SEN, and SPE were defined as follows:

(12) $$\mathrm{ACC}=\frac{TP+TN}{TP+TN+FP+FN};$$

(13) $$\mathrm{SEN}=\frac{TP}{TP+FN};$$

(14) $$\mathrm{SPE}=\frac{TN}{TN+FP}$$

where *TP* represents true positive classifications, *TN* represents true negative classifications, *FP* represents false positive classifications, and *FN* represents false negative classifications.

## 3. Results

This section describes the results of 2D CNN model-based liver ROI segmentation (Section 3.1) and 1D CNN model-based liver fibrosis stage classification (Section 3.2).

### 3.1. 2D CNN Model-Based Liver ROI Segmentation

**Figure 6:**
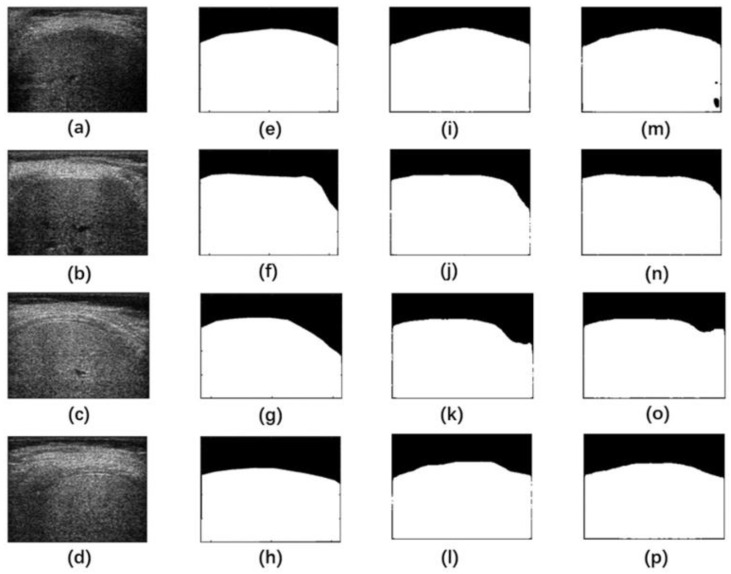
Representative liver ROI segmentation results on the test set in Group A by U-Net and Attention U-Net. (**a**–**d**) Liver B-mode ultrasound images, (**e**–**h**) manual delineation as the reference standard, (**i**–**l**) segmentation images by Attention U-Net, (**m**–**p**) segmentation images by U-Net. The white pixels in the segmentation images represented the segmented liver region, and the black pixels represented the segmented non-liver region. ROI: region of interest.

Figure 6 shows representative liver ROI segmentation results on the test set in Group A by the U-Net and Attention U-Net models. The segmentation performance of the U-Net model at the boundary was relatively lower, even with some missing boundaries. The Attention U-Net model performed better in liver ROI segmentation and was closer to the reference standard than the U-Net model. This indicated that the attention mechanism enhanced the model’s capability for processing details.

**Figure 7:**
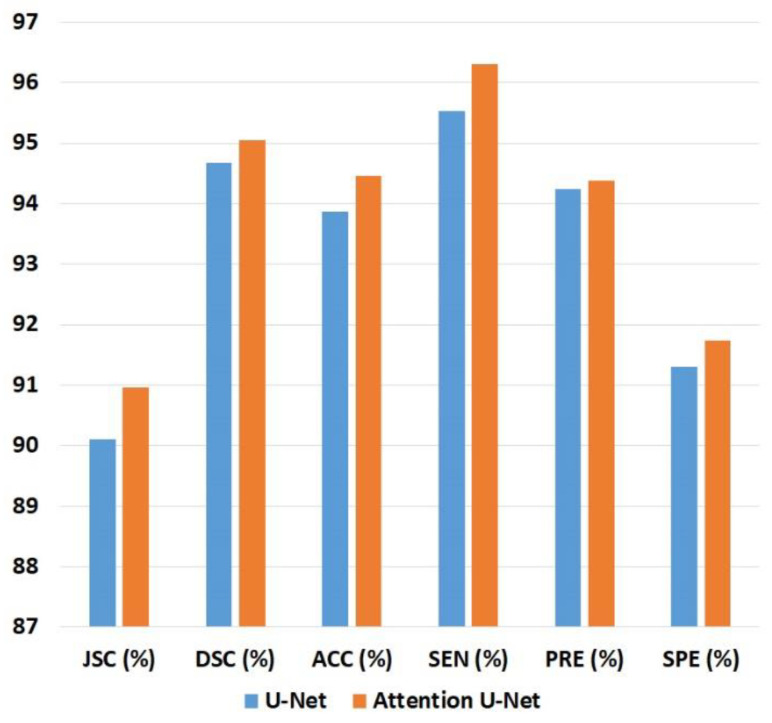
Bar chart of the results in Table 1. JSC: Jaccard similarity coefficient; DSC: Dice similarity coefficient; ACC: accuracy; SEN: sensitivity; PRE: precision; SPE: specificity.

Table 1 and Figure 7 show the liver ROI segmentation performance on the test sets in Group A by the U-Net and Attention U-Net models in terms of JSC, DSC, ACC, SEN, PRE, and SPE. The Attention U-Net model was slightly better than the U-Net model in each metric. Compared with the U-Net model, the Attention U-Net model was 0.85% higher in JSC, 0.37% higher in DSC, 0.78% higher in ACC, 0.77% higher in SEN, 0.14% higher in PRE, and 0.42% higher in SPE. Because of its better segmentation performance, the Attention U-Net model was chosen as the final 2D CNN model for detecting liver ROIs in Group B.

### 3.2. 1D CNN Model-Based Liver Fibrosis Stage Classification

After data augmentation on the spectrum signals in Group B, the numbers of spectrum signal samples in the training set, validation set, and test set for different liver fibrosis stage classifications are shown in Table 2. Note that the numbers in Table 2 applied to each of the three kinds of frequency spectra, i.e., amplitude, phase, and power. The results of liver fibrosis stage classification for each kind of spectra are described in the following subsections.

#### 3.2.1. Liver Fibrosis Stage Classification for Amplitude Spectra

**Figure 8:**
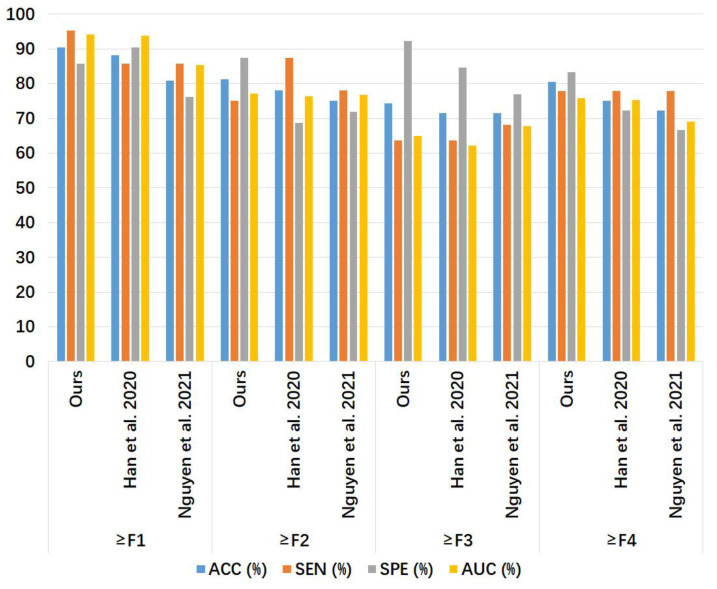
Bar chart of the results in Table 3. The compared models were Han et al. [7] and Nguyen et al. [8]. ACC: accuracy; SEN: sensitivity; SPE: specificity; AUC: area under the receiver operating characteristic curve.

Table 3 and Figure 8 show the liver fibrosis stage classification performance on the amplitude spectrum test set in Group B by the proposed method, compared to that by the 1D CNN models of Han et al. [7] and Nguyen et al. [8]. For ≥F1, our model had larger ACC, SEN, and AUC values than the other two models and an SPE value larger than Nguyen et al.’s model [8] but lower than Han et al.’s model [7]. For ≥F2, the SEN value of our model was lower than that of the other two models, and the values of the other metrics of our model were slightly higher. For ≥F3, Nguyen et al.’s model [8] had a higher AUC value, and our model outperformed the other two models in ACC, SEN, and SPE. For ≥F4, our model outperformed the other two models in ACC, SPE, and AUC, while the SEN of our model was the same as the other two models.

#### 3.2.2. Liver Fibrosis Stage Classification for Phase Spectra

**Figure 9:**
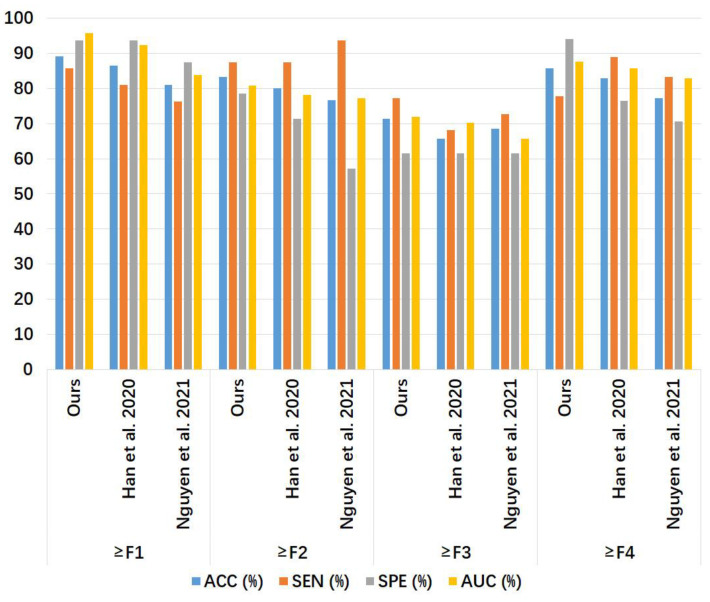
Bar chart of the results in Table 4. The compared models were Han et al. [7] and Nguyen et al. [8]. ACC: accuracy; SEN: sensitivity; SPE: specificity; AUC: area under the receiver operating characteristic curve.

Table 4 and Figure 9 show the liver fibrosis stage classification performance on the phase spectrum test set by the proposed method, compared to that by the 1D CNN models of Han et al. [7] and Nguyen et al. [8]. For ≥F1, our model had larger values in ACC and SEN than the other two models, while the SPE of our model was equal to Han et al.’s model [7] and higher than Nguyen et al.’s model [8]. For ≥F2, our model outperformed the other two models in ACC, SPE, and AUC, with an SEN value equal to Han et al.’s model [7] and lower than Nguyen et al.’s model [8]. For ≥F3, our model outperformed the other two models in ACC, SEN, and AUC, with the same SPE value as the other two models. For ≥F4, our model had higher values in ACC, SPE, and AUC than the other two models, with an SEN value lower than the other two models.

#### 3.2.3. Liver Fibrosis Stage Classification for Power Spectra

**Figure 10:**
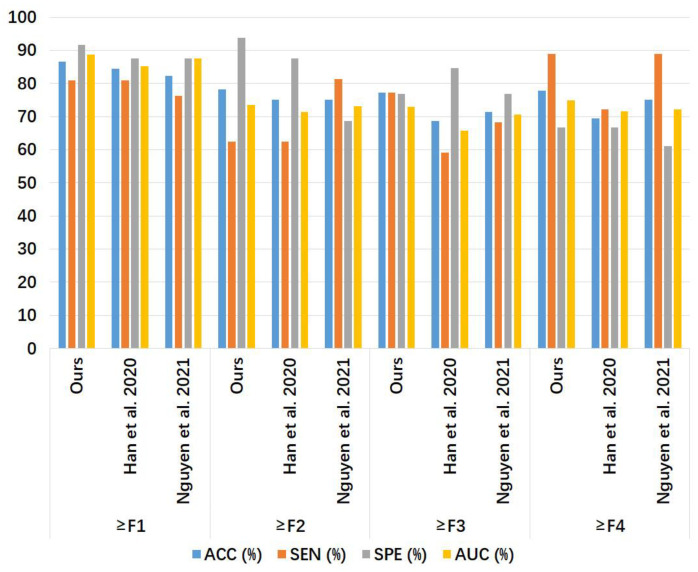
Bar chart of the results in Table 5. The compared models were Han et al. [7] and Nguyen et al. [8]. ACC: accuracy; SEN: sensitivity; SPE: specificity; AUC: area under the receiver operating characteristic curve.

Table 5 and Figure 10 show the liver fibrosis stage classification performance on the power spectrum test set by the proposed method, compared to that of the 1D CNN models of Han et al. [7] and Nguyen et al. [8]. For ≥F1, the values of ACC, SPE, and AUC of our model were higher than those of the other two models with the same SEN. For ≥F2, our model had higher values in ACC, SPE, and AUC than the other two models, with an SEN value lower than Nguyen et al.’s model [7] and equal to Han et al.’s model [8]. For ≥F3, our model had ACC, SEN, and AUC values higher than the other two models, with an SPE value lower than Han et al.’s model [7] and equal to Nguyen et al.’s model [8]. For ≥F4, our model had ACC and AUC values higher than the other two models, with an SEN value equal to Nguyen et al.’s model [8] and higher than Han et al.’s model [7], and with an SPE value equal to Han et al.’s model [7] and higher than Nguyen et al.’s model [8].

## 4. Discussion

In this study, we proposed a two-step approach for assessing liver fibrosis using deep learning models applied to ultrasound RF signals. First, 2D CNNs (U-Net and Attention U-Net) were used for automatic liver ROI segmentation from B-mode ultrasound images reconstructed from the RF signals. Second, 1D CNNs were utilized for liver fibrosis stage classification based on frequency spectra (amplitude, phase, and power) of the segmented ROI signals. Ultrasound RF signals collected from 613 participants were analyzed for liver ROI segmentation and those from 237 participants for liver fibrosis stage classification. Experimental results demonstrated the feasibility of both the 2D and 1D CNNs in liver parenchyma detection and liver fibrosis characterization. The findings of this study shed light on deep learning analysis of ultrasound RF signals in the frequency domain with automatic ROI segmentation.

In the context of ultrasound techniques for liver fibrosis assessment, B-mode ultrasound imaging is the most widely used technique in clinical settings. However, it is qualitative in nature and can be affected by post-processing parameters such as the dynamic range. Ultrasound elastography techniques have been used to quantitatively stage liver fibrosis by measuring the strain, stiffness, or shear wave speed of the liver tissue [29,30], but these techniques require specialized ultrasound scanners, and the measurement can be affected by hepatic inflammation [31,32].

Ultrasound techniques on the basis of analyzing the ultrasound RF signals may be compatible with most ultrasound scanners, as B-mode ultrasound images are constructed using the envelopes of RF signals. Although quantitative ultrasound techniques [4] can be utilized to assess liver fibrosis, they usually need specific mathematical or physical models under specific model assumptions to extract a single feature parameter each time.

Deep learning analysis of ultrasound RF signals is emerging for tissue characterization, as it can automatically extract multi-level information as feature parameters through CNNs [7,8,9,10,11,12,13]. In 2020, Han et al. [7] proposed 1D CNNs based on ultrasound RF signals to assess hepatic steatosis in 204 adults, with a 96% accuracy on the test set. In 2021, Nguyen et al. [8] presented ultrasound RF signal-based 1D CNNs to evaluate hepatic steatosis in 52 rabbits, with a 74% accuracy on the test set. In 2021, Cheng et al. [9] introduced 1D bidirectional recurrent neural networks based on ultrasound RF signals to analyze liver fibrosis in 160 rats, with an 80% accuracy on the test set. In 2022, Luo et al. [10] presented multichannel CNNs based on ultrasound RF signals to assess osteoporosis in 274 participants, with an 83.05% accuracy, higher than the accuracy by the conventional speed of sound method (66.67%). In 2022, Sanabria et al. [11] employed 1D, 2D, and three-dimensional CNNs based on ultrasound RF signals and their frequency spectra (power and phase) to assess hepatic steatosis in 31 patients. In 2022, Huang et al. introduced 1D CNNs based on ultrasound RF signals to classify 230 adults’ liver fibrosis stages [12]. In 2023, Xie et al. proposed deep learning models based on ultrasound RF signals for rapid intraoperative multi-molecular diagnosis of glioma [13]. Currently, there are only two studies [9,12] involving deep learning models based on ultrasound RF signals to evaluate liver fibrosis stages. However, both Cheng et al. [9] and Huang et al. [12] only used the time-domain information of ultrasound RF signals to train and test the deep learning models, but they did not investigate the feasibility of deep learning models based on frequency-domain information of ultrasound RF signals in liver fibrosis evaluation. Furthermore, the liver ROIs were manually delineated by Cheng et al. [9] and Huang et al. [12], lacking automatic ROI identification. In this work, we proposed a new strategy for deep learning characterization of liver fibrosis based on RF signals, with 2D CNNs for automatic liver ROI segmentation and 1D CNNs for liver fibrosis stage classification based on frequency spectra of the segmented ROI signals.

The performance of the 2D CNN models in segmenting liver ROIs from reconstructed B-mode ultrasound images was compared in terms of metrics, including DSC and JSC. Note that DSC and JSC have been frequently used in the medical image segmentation field to quantitatively evaluate the segmentation performance. A higher value closer to 1 indicates a better segmentation result, meaning that the segmentation was closer to the reference standard. The results in Figure 6 and Table 1 indicated that both the U-Net and Attention U-Net models were feasible for automatic liver ROI segmentation in B-mode ultrasound images. The attention mechanism adopted in the Attention U-Net model improved the segmentation performance over the U-Net model. However, the average training time per epoch for the U-Net model (121.84 s) was less than that for the Attention U-Net model (139.98 s), indicating that the attention mechanism also increased computational cost.

The performance of our 1D CNN models in classifying liver fibrosis stages was compared with the 1D CNN models by Han et al. [7] and Nguyen et al. [8] in terms of AUC, ACC, SEN, and SPE. Each of the four metrics had a maximum value of 1 (or 100%). A larger value of AUC corresponded to a higher diagnostic value. A larger value of ACC corresponded to a more accurate diagnosis, i.e., a higher rate of true positive and true negative predictions in the total samples [Equation (12)]. A larger value of SEN corresponded to a lower rate of missed diagnosis [Equation (13)]. A larger value of SPE corresponded to a lower rate of misdiagnosis [Equation (14)]. The results in Table 3, Table 4 and Table 5 indicated that our 1D CNN models based on ROI spectrum signals were feasible for liver fibrosis stage classification. From Table 3, it can be seen that our model based on amplitude spectra had better liver fibrosis stage classification performance than the 1D CNN models of Han et al. [7] and Nguyen et al. [8] for ≥ F4 in terms of AUC, ACC, SEN, and SPE. From Table 4, it can be seen that our model based on phase spectra outperformed the other two models for ≥ F1 and ≥ F3 in terms of AUC, ACC, SEN, and SPE. From Table 5, it can be seen that our model based on power spectra outperformed the other two models for ≥F1 and ≥F4 in terms of AUC, ACC, SEN, and SPE.

**Figure 11:**
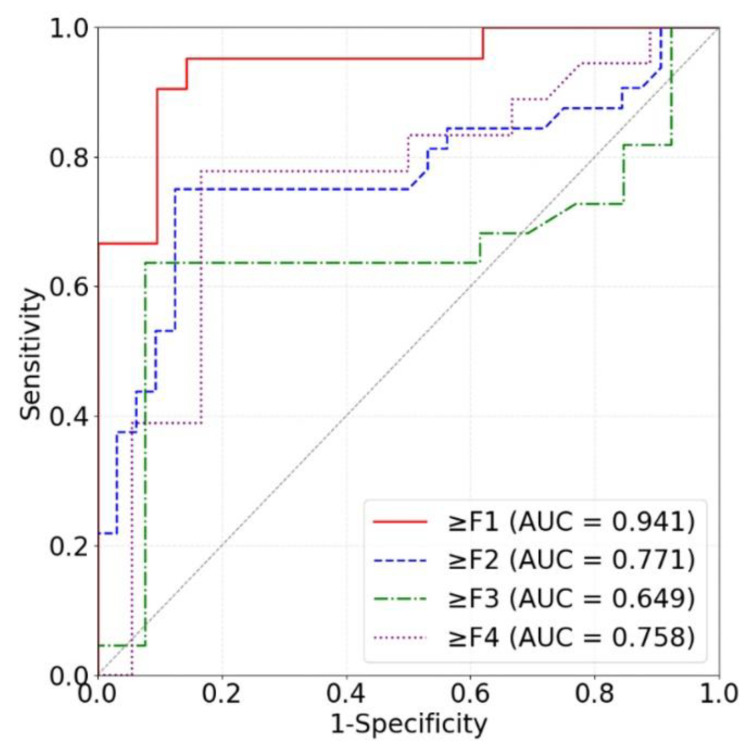
ROC curves of the proposed method based on amplitude spectra for classifying liver fibrosis stage ≥F1 (red), ≥F2 (blue), ≥F3 (green), and ≥F4 (purple). ROC: receiver operating characteristic; AUC: area under the ROC curve.

**Figure 12:**
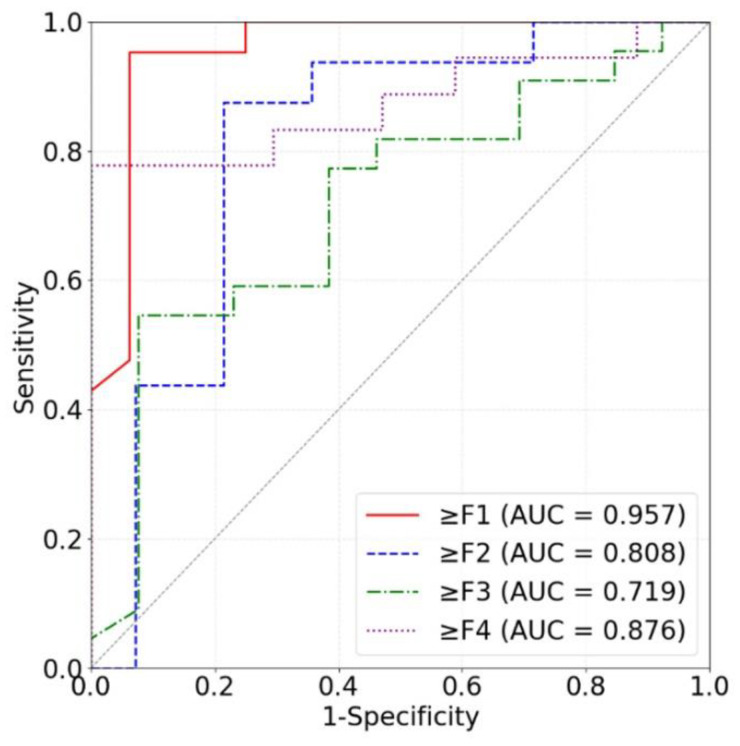
ROC curves of the proposed method based on phase spectra for classifying liver fibrosis stage ≥F1 (red), ≥F2 (blue), ≥F3 (green), and ≥F4 (purple). ROC: receiver operating characteristic; AUC: area under the ROC curve.

**Figure 13:**
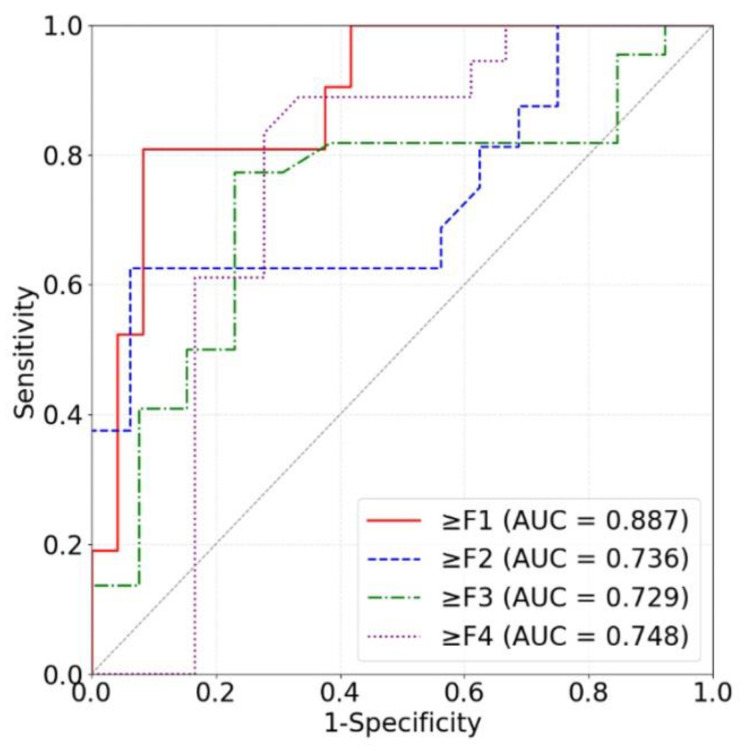
ROC curves of the proposed method based on power spectra for classifying liver fibrosis stage ≥F1 (red), ≥F2 (blue), ≥F3 (green), and ≥F4 (purple). ROC: receiver operating characteristic; AUC: area under the ROC curve.

Our models based on the three kinds of frequency spectra were compared in terms of different liver fibrosis stage classifications (Figure 11, Figure 12 and Figure 13). For ≥F1, ≥F2, and ≥F4, our model based on phase spectra all yielded the highest AUC values of 0.957, 0.808, and 0.876, respectively. For ≥ F3, our model based on power spectra had the highest AUC of 0.729. Overall, the performance of the proposed deep learning method was the best when using phase spectrum signals for ≥F1 (AUC: 0.957; ACC: 89.19%; SEN: 85.17%; SPE: 93.75%), ≥F2 (AUC: 0.808; ACC: 83.34%; SEN: 87.50%; SPE: 78.57%), and ≥F4 (AUC: 0.876; ACC: 85.71%; SEN: 77.78%; SPE: 94.12%). The classification performance of our method was the best when using power spectrum signals for ≥ F3 (AUC: 0.729; ACC: 77.14%; SEN: 77.27%; SPE: 76.92%). In particular, the proposed method based on ROI phase spectrum signals was recommended for liver fibrosis stage classification, especially for early fibrosis detection (≥F1).

This study has limitations. First, the sample size and diversity of the dataset were limited. The clinical data were collected from a single center with a single ultrasound scanner. Second, the proposed method yielded satisfying performance in liver fibrosis stage classification for ≥ F1, ≥ F2, and ≥ F4 (all AUCs > 0.80) when using ROI phase spectrum signals, but the performance for ≥ F3 (AUC: 0.719) was lower. In future work, more ultrasound RF data may be collected to further validate the performance of the proposed method, the cross-center and cross-scanner performance of the proposed method may be evaluated, and the performance for ≥ F3 may be improved.

## 5. Conclusions

In this study, we proposed an approach for liver fibrosis assessment using deep learning models on ultrasound RF signals. The proposed method consisted of 2D CNNs for automatic liver ROI segmentation from reconstructed B-mode ultrasound images and 1D CNNs for liver fibrosis stage classification based on frequency spectra (amplitude, phase, and power) of the segmented ROI signals. Experimental results demonstrated the feasibility of both the 2D and 1D CNNs in liver parenchyma detection and liver fibrosis characterization. The proposed methods have provided a new strategy for liver fibrosis assessment based on ultrasound RF signals. In particular, the proposed method based on ROI phase spectrum signals was recommended for liver fibrosis stage classification, especially for early fibrosis detection. The findings of this study shed light on deep learning analysis of ultrasound RF signals in the frequency domain with automatic ROI segmentation.

## Figures and Tables

**Table 1:** Liver ROI segmentation performance on the test sets in Group A by U-Net and Attention U-Net in terms of JSC, DSC, ACC, SEN, PRE, and SPE. The larger value of each metric is indicated as bold numbers. JSC: Jaccard similarity coefficient; DSC: Dice similarity coefficient; ACC: accuracy; SEN: sensitivity; PRE: precision; SPE: specificity. Attention U-Net was slightly better than U-Net on each metric.

Metrics	U-Net	Attention U-Net
JSC (%)	90.11	**90.96**
DSC (%)	94.68	**95.05**
ACC (%)	93.86	**94.46**
SEN (%)	95.53	**96.30**
PRE (%)	94.24	**94.38**
SPE (%)	91.31	**91.73**

**Table 2:** Numbers of spectrum signal samples in the training set, validation set, and test set in Group B for different liver fibrosis stage classifications. Each sample was sized 768 points × 256 lines.

Fibrosis Stage	Training Sets	Validation Sets	Test Sets
≥F1	315	42	42
≥F2	248	32	33
≥F3	277	35	35
≥F4	284	36	39

**Table 3:** Liver fibrosis stage classification performance on the amplitude spectrum test set in Group B by the proposed method, compared to that by the 1D CNN models of Han et al. [7] and Nguyen et al. [8]. The largest value of each metric for each classification is indicated as bold numbers. ACC: accuracy; SEN: sensitivity; SPE: specificity; AUC: area under the receiver operating characteristic curve.

Fibrosis Stage	Model	ACC (%)	SEN (%)	SPE (%)	AUC
	Ours	**90.48**	**95.24**	85.71	**0.941**
≥F1	Han et al. [7]	88.10	85.71	**90.48**	0.937
	Nguyen et al. [8]	80.95	85.71	76.19	0.853
	Ours	**81.25**	75.00	**87.50**	**0.771**
≥F2	Han et al. [7]	78.13	**87.50**	68.75	0.763
	Nguyen et al. [8]	75.00	78.13	71.88	0.767
	Ours	**74.29**	63.64	**92.31**	0.649
≥F3	Han et al. [7]	71.43	63.64	84.62	0.621
	Nguyen et al. [8]	71.43	**68.18**	76.92	**0.678**
	Ours	**80.56**	**77.78**	**83.33**	**0.758**
≥F4	Han et al. [7]	75.00	**77.78**	72.22	0.753
	Nguyen et al. [8]	72.22	**77.78**	66.67	0.691

**Table 4:** Liver fibrosis stage classification performance on the phase spectrum test set in Group B by the proposed method, compared to that by the 1D CNN models of Han et al. [7] and Nguyen et al. [8]. The largest value of each metric for each classification is indicated as bold numbers. ACC: accuracy; SEN: sensitivity; SPE: specificity; AUC: area under the receiver operating characteristic curve.

Fibrosis Stage	Model	ACC (%)	SEN (%)	SPE (%)	AUC
	Ours	**89.19**	**85.71**	**93.75**	**0.957**
≥F1	Han et al. [7]	86.49	80.95	93.75	0.923
	Nguyen et al. [8]	81.08	76.19	87.50	0.839
	Ours	**83.34**	87.50	**78.57**	**0.808**
≥F2	Han et al. [7]	80.00	87.50	71.43	0.781
	Nguyen et al. [8]	76.67	**93.75**	57.14	0.772
	Ours	**71.43**	**77.27**	**61.54**	**0.719**
≥F3	Han et al. [7]	65.71	68.18	**61.54**	0.703
	Nguyen et al. [8]	68.57	72.73	**61.54**	0.657
	Ours	**85.71**	77.78	**94.12**	**0.876**
≥F4	Han et al. [7]	82.86	**88.89**	76.47	0.858
	Nguyen et al. [8]	77.14	83.33	70.59	0.828

**Table 5:** Liver fibrosis stage classification performance on the power spectrum test set in Group B by the proposed method, compared to that by the 1D CNN models of Han et al. [7] and Nguyen et al. [8]. The largest value of each metric for each classification is indicated as bold numbers. ACC: accuracy; SEN: sensitivity; SPE: specificity; AUC: area under the receiver operating characteristic curve.

Fibrosis Stage	Model	ACC (%)	SEN (%)	SPE (%)	AUC
	Ours	**86.67**	**80.95**	**91.67**	**0.887**
≥F1	Han et al. [7]	84.44	**80.95**	87.50	0.852
	Nguyen et al. [8]	82.22	76.19	87.50	0.875
	Ours	**78.13**	62.50	**93.75**	**0.736**
≥F2	Han et al. [7]	75.00	62.50	87.50	0.713
	Nguyen et al. [8]	75.00	**81.25**	68.75	0.732
	Ours	**77.14**	**77.27**	76.92	**0.729**
≥F3	Han et al. [7]	68.57	59.09	**84.62**	0.657
	Nguyen et al. [8]	71.43	68.18	76.92	0.706
	Ours	**77.78**	**88.89**	**66.67**	**0.748**
≥F4	Han et al. [7]	69.44	72.22	**66.67**	0.715
	Nguyen et al. [8]	75.00	**88.89**	61.11	0.721

## Data Availability

The ultrasound radiofrequency data may be provided upon reasonable requests for scientific research purposes.
